# Determinants of the quality of life amongst HIV clinic attendees in Malang, Indonesia

**DOI:** 10.1186/s12889-021-11321-7

**Published:** 2021-06-30

**Authors:** Sri Sunaringsih Ika Wardojo, Ya-Li Huang, Kun-Yang Chuang

**Affiliations:** 1 School of Public Health, Taipei Medical University, Taipei, Taiwan; 2 Faculty of Health Science, University of Muhammadiyah, Malang, Indonesia; 3 Department of Public Health, School of Medicine, College of Medicine, Taipei Medical Univeristy, Taipei, Taiwan

**Keywords:** QoL, HIV, Stigma, ART adherence

## Abstract

**Background:**

As the number of people living with human immunodeficiency virus (HIV; PLHIV) in Indonesia has increased in recent years, more efforts have been expended to improve their health status. However, in a country where PLHIV are very much stigmatized, there has been little research concerning their quality of life (QoL). Hence, this study aimed to assess the QoL among PLHIV and its associated factors. Findings of this research can contribute to improving the health and wellbeing of PLHIV in Indonesia.

**Methods:**

A cross-sectional survey with convenience sampling was conducted from June to September 2018, at four healthcare centers in Malang, Indonesia. PLHIV, aged 18 years or over, were asked if they would like to participate in this study when they came to a health center to receive services. To protect confidentiality, the healthcare staff at the clinics assisted with recruitment and face-to-face interviews with structured questionnaires. Measurements included sociodemographic, medication-related, social support, HIV-stigma, and QoL variables.

**Results:**

In total, 634 PLHIV agreed to participate in this study. A multivariate linear regression analysis showed that being older, having a job, living in an urban area, having better access to healthcare services, adhering to medication, being in an antiretroviral therapy (ART) program for more than 1 year, experiencing a lower level of stigma, and receiving more social support were associated with a better QoL. The regression model had an adjusted *R*^2^ of 0.21.

**Conclusions:**

Findings from this research have significant policy implications. Policies focused on reducing social stigma and promoting medication adherence will likely have a positive impact on the QoL of PLHIV. Increasing public awareness and acceptance of PLHIV in Indonesia remains challenging, but would likely have significant impacts. Furthermore, interventions should also focus on reducing disparities in QoL between PLHIV living in rural areas and those in urban areas.

## Background

Indonesia, ranked 13th worldwide in the number of people living with human immunodeficiency virus (HIV; PLHIV), with an estimates of PLHIV in 2020 were 543,075 [1], and the annual incident cases of HIV in Indonesia has also increased from 12,214 per year in 2013 to 50,282 per year in 2020 [1]. The first AIDS national strategy was implemented in 1997 [2], with a focus on HIV/AIDS control through care and treatment support programs. In 2003, Indonesia Ministry of Health implemented a second AIDS national strategy which is still in effect in 2021, with its main focus being HIV prevention and treatment [3]. Antiretroviral Treatment (ART) is being fully subsidized and is provided at primary health care centers. This guidelines recommended immediate initiation of ART after HIV diagnosis regardless of the individual’s clinical or immunological status [3]. Other programs such as needle exchange program and methadone maintenance treatment were implemented as well. As of September 2020, there were 1515 ART primary care centers nationwide [4].

In Indonesia, with the largest Muslim population in the world, communicating about sex and HIV are often considered taboo, and this leads to a low understanding of the symptoms and preventive measures of this disease [5]. Moreover, social stigmatization and discrimination against PLHIV still pose significant obstacles to prevention and treatment [6–8]. As such, previous studies [7, 8] reported that PLHIV have experienced rejection by healthcare workers, have been denied care because of their HIV status, and thus fear to disclose their status. Lack of family, peer, and community support also contributes to high loss of follow-up of ART medication [9]. All of these have negative impacts on the wellbeing of PLHIV. Quality of life (QoL) is an important component when evaluating the wellbeing of PLHIV, and despite the recent growth in numbers, little research has been conducted concerning the QoL among PLHIV. Moreover, as ART has been available as long-term regimen care for PLHIV, the QoL has become a significant indicator for evaluating the impacts of ART [10]. Hence, research on QoL among PLHIV will provide additional insights into challenges they face, and into how to improve their wellbeing.

Previous research, using the World Health Organization Quality of Life in the HIV-infected person instrument (WHOQOL-HIV-BREF) questionnaire [11], indicated that a higher QoL level was associated with being male [12, 13], being of a younger age [14], being employed [15, 16], and having a better financial status and a higher educational level [17–21]. Moreover, factors such as adherence to ART [22] and social support [23] were also associated with a higher level of QoL, while poor access to healthcare services [24] and stigmatization [16, 25] were associated with a lower QoL.

PLHIV in Indonesia face several challenges, such as high levels of community stigmatization and discrimination, and they were reported to have a lower level of QoL compared to those in other Asian countries [21, 26]. Hence, this study aimed to assess the QoL among PLHIV and whether sociodemographic, social support, stigmatization, or healthcare factors were associated with QoL. To improve the life and wellbeing of PLHIV in Indonesia, it is essential to understand factors associated with their QoL before appropriate interventions can be devised.

## Methods

### Study design and participants

A cross-sectional study with a convenience sampling method was conducted. Time and sample size concerns were the reasons for recruiting PLHIV from clinics. PLHIV faces prejudice and negative consequences of social isolation and exclusion once identity is known. Surveys utilizing snowball sampling, or other form of random sampling, would require a long period of time to build up trust with participants, and may also have the problem of having a small sample size, as well. Study participants were recruited from four clinics (Puskesmas Dinoyo, Puskesmas Kendalsari, RST Dr. Soepraoen, and RS Islam Malang) which provide voluntary counseling and testing (VCT) and ART services in Malang, Indonesia. Malang was reported to have the second-highest HIV+ prevalence rate in East Java Province [27].

To protect the confidentiality of PLHIV, healthcare staff at the clinics assisted with recruiting participants and data collection. Because participants were concerned with privacy and disclosure, and were less interested in meeting with outside researchers, they were more willing to participate in surveys if administered by clinic staff, with whom they have regular contact. Staff at the clinics received training from the researchers regarding the procedures of recruitment and data collection. From June to September 2018, PLHIV who came to the clinics to receive health services were asked if they would be willing to participate in this study. Only those aged over 18 years were recruited. Face-to-face interviews were then conducted with a structured questionnaire by the same healthcare staff. In total, 634 HIV patients agreed to participate in this study.

### Measurements

Variables used in this study included QoL, social support, and stigma measures, as well as sociodemographic characteristics and medication-related variables. QoL was measured using the WHOQOL-HIV BREF questionnaire. The WHOQOL-HIV BREF consists of 31 items rated on a 5-point Likert scale, which cover six domains of physical, psychological, level of independence, social relationship, environment, and spirituality. A total score is computed, with a higher score indicating a better QoL [28, 29]. This instrument was developed and validated in several countries [17, 30–32], including Indonesia [26, 33]. The reliability of this instrument was high, with α = 0.86 [33].

Social support was measured using the Multidimensional Scale of Perceived Social Support (MSPSS) [34]. It covers support in time of need from friends, family, and significant others. The MSPSS consists of 12 items, such as “I can talk about my problems with my family” and “My friends really try to help me”, that ask respondents to rate their perceived level of support on a Likert scale ranging from 1 “very strongly disagree” to 7 “very strongly agree”, with a higher score indicating greater social support. A previous study reported that the Indonesian version of the MSPSS had a reliability of 0.81 [35].

The HIV stigma scale by Reinius et al. [36], a version modified from Berger et al. [37], was used to measure stigma. It consists of 12 items with a Likert scale, covering four areas of stigma: personalized, disclosure, public attitudes, and self-image. Each subscale consists of three items, and the response to each item ranges from 1 “strongly agree” to 4 “strongly disagree”. The total score in each subscale is calculated by adding up the raw values of the items, and a higher score reflects a higher level of HIV-related stigmatization. Sample items include “People I care about stopped calling after learning I have HIV” and “I have been hurt by how people reacted to learning I have HIV”. It previously demonstrated high reliability and validity among PLHIV, with reliability for the entire scale of 0.83 [38].

Sociodemographic characteristics included age, sex (male or female), marital status (unmarried, married, divorced, or widowed), educational level (primary education, secondary education, or tertiary education), working status (yes or no), household monthly income (<US$108, US$108 ~ 180, US$180 ~ 250, and > US$250, with the average exchange rate in 2018 of US$1.00 ≈ Rp14,000), and living area (urban or rural). Accessibility to health services was assessed with the question “Thinking of access of healthcare services in the past 12 months, how accessible was it for you to obtain the healthcare services you needed?” [39]. The response categories were ‘very difficult’, ‘difficult’, ‘average’, ‘easy’, and ‘very easy’. This question did not specifically refer to time, cost, or other barriers to health services, but mainly tried to assess the subjective feeling of how difficult it was to obtain needed services. In the data analysis, “very difficult” and “difficult” were grouped together to represent “difficult”, “average’ and ‘easy’ were merged together to represent “easy’, while “very easy” stands by itself.

Medication-related variables included the cluster difference 4 (CD4) count (< 200, 200 ~ 500, or > 500 cells/mm^3^), length of ART (< 2 or ≥ 2 years), length of diagnosis with HIV counted in years since the first diagnosis of HIV, and adherence to medication (yes or no). Adherence to medication was measured using the AIDS (acquired immunodeficiency syndrome) Clinical Trial Group (ACTG) questionnaire [40–42], which assesses the number of missed pills (at least one dose) in the past 2 weeks, as being non-adherent to ART with a yes/no answer.

### Statistical analysis

Descriptive univariate statistics consisted of the frequency and percentage for categorical variables, and the mean and standard deviation (SD) for continuous variables. A *t*-test, analysis of variance (ANOVA), and Pearson’s correlation, depending on the type of variable, were used to assess the relationship between two variables. A multivariate linear regression was used to assess relationships between predictors and QoL. The level of statistical significance was set to *p* < 0.05. All analyses were performed using SPSS vers. 20 (SPSS, Chicago, IL, USA).

### Ethical considerations

Ethical clearance for this study was obtained and approved by the University of Muhammadiyah Malang Ethics Committee of Indonesia (no E.5.1/066/KEPK-UMM/II/2018). As all participants were adults, the risks of participation in this study were explained to participants before they completed the written consent form.

## Results

Table 1 shows the basic demographic characteristics and QoL scores. In total, 634 PLHIV participated in this study. Nearly 40% of them were younger than 30 years, and 77% of them were males. As to medication variables, 70% of participants had not missed their medication in the past 2 weeks, and 52.5% had been on ART for more than 2 years. Higher QoL scores were found among participants aged 61 ~ 65 and 51 ~ 55 years. Factors that may be associated with higher QoL scores were being married, working, living in an urban area, having access to health care, adhering to medication, and being on ART for more than 2 years.

**Table 1:** Basic characteristics of participants (*N* = 634)

	***n***	%	QoL
			Mean (SD)
Age (years)*			
< 20	40	6.3	84.81 (7.69)
21–25	82	12.9	82.02 (7.49)
26–30	130	20.5	84.38 (6.93)
31–35	108	17.0	86.72 (8.16)
36–40	70	11.0	84.94 (6.51)
41–45	82	12.9	84.97 (6.69)
46–50	57	9.0	85.57 (9.60)
51–55	32	5.0	87.54 (6.20)
56–60	26	4.1	84.98 (7.23)
61+	7	1.1	87.90 (4.03)
Sex			
Male	488	77.0	84.78 (7.76)
Female	146	23.0	85.60 (6.68)
Educational level			
Primary education	182	28.7	85.01 (6.66)
Secondary education	238	37.5	84.61 (7.18)
Tertiary education	214	33.8	85.33 (8.54)
Marital status*			
Unmarried	282	44.5	83.96 (7.17)
Married	230	36.3	86.01 (8.24)
Divorced	90	14.2	84.83 (5.17)
Widowed	32	5.0	86.83 (9.60)
Household income			
< US$ 108	144	22.7	84.54 (7.78)
US$ 108 ~ US$ 180	258	40.7	84.86 (7.90)
US$ 180 ~ US$ 250	142	22.4	84.76 (6.28)
> US$ 250	90	14.2	86.29 (7.79)
Working*			
No	83	13.1	82.73 (9.23)
Yes	551	86.9	85.31 (7.18)
Living area**			
Urban	291	45.9	86.01 (7.69)
Rural	343	54.1	84.09 (7.28)
Accessibility for obtaining healthcare services**			
Difficult	160	25.2	84.28 (6.09)
Easy	314	49.5	84.37 (7.60)
Very easy	160	25.2	86.85 (8.36)
Adherence to medication***			
Yes	446	70.3	85.71 (7.54)
No	188	29.7	83.22 (7.21)
CD4 count (cells/mm^3^)			
< 200	210	33.1	84.31 (7.77)
200 ~ 500	335	52.8	85.34 (6.94)
> 500	89	14.1	85.11 (8.92)
Length of ART (years)***			
≤ 1.99	301	47.5	83.77 (7.55)
2 ~ 14.1	333	52.5	86.05 (7.35)

* *p* < 0.05; ** *p* < 0.01; *** *p* < 0.001

*QoL* quality of life; *SD* standard deviation; *CD4* cluster difference 4; *ART* adherence to antiretroviral therapy

The average exchange rate in 2018 was US$1.00 ≈ Rp 14,000

Table 2 shows correlations of social support, stigma subscales, and length of the diagnosis with the QoL. Results indicated that the QoL had a positive and significant correlation with social support, and had negative and significant correlations with all stigma subscales (personalized, disclosure, and self-image). Correlations ranged 0.08 ~ 0.25. The correlation between the length of time with an HIV diagnosis and the QoL was not statistically significant.

**Table 2:** Correlation coefficients among social support, stigma, length of diagnosis and quality of life (QoL)

	QoL	Social support	Stigma: Personalized	Stigma: disclosure	Stigma: public attitudes	Stigma: self-image	Length of diagnosis with HIV
QoL	1						
Social support	0.24^**^	1					
Stigma: personalized	−0.08^*^	0.22	1				
Stigma: disclosure	−0.14**	− 0.03	0.002	1			
Stigma: public attitudes	−0.10**	− 0.29	− 0.25^**^	0.15^**^	1		
Stigma: self-image	− 0.25**	0.01	− 0.07^*^	0.09^*^	0.13^**^	1	
Length of diagnosis with HIV	0.06	−0.08*	0.01	− 0.09*	− 0.03	−0.04	1

* *p* < 0.05; ** *p* < 0.01

*HIV* human immunodeficiency virus

Table 3 shows results of the multivariate linear regression. After controlling for other variables, age, working status, living area, access to healthcare, medication, length of ART, personalized stigma, disclosure stigma, self-image stigma, and social support were significantly associated with the QoL. The regression model had an adjusted *R*^2^ of 0.21.

**Table 3:** Multivariate linear regression of the quality of life (QoL)

	Unstandardized coefficients		Standardized coefficients
	B	SE	β
**Sociodemographic characteristics**			
Age	0.27	0.13	0.08*
Sex	0.75	0.65	0.04
Educational level	0.61	0.35	0.06
Marital Status (ref: Unmarried)			
a. married	1.03	0.64	0.06
b. divorced	−0.05	0.86	−0.003
c. widowed	0.58	1.30	0.01
Household income	−0.00	0.29	0.00
Working (ref: No)	1.70	0.83	0.07*
Living area (ref: Urban)	−1.74	0.54	−0.11**
Accessibility of health care (ref: Difficult)			
a. easy	0.92	0.38	0.08**
b. very easy	0.94	0.36	0.04*
**Medication variables**			
Adherence to medication	1.99	0.61	0.12**
CD4 count	−0.17	0.41	−0.01
Length of ART (ref: ≤1.99)	1.95	0.74	0.13**
Length of diagnosis with HIV	−0.13	0.14	−0.05
**Stigma subscales**			
Stigma: personalized	−0.61	0.18	−0.13**
Stigma: disclosure	−0.34	0.17	− 0.07*
Stigma: public attitudes	− 0.14	0.16	− 0.03
Stigma: self-image	−1.05	0.19	− 0.19***
**Social support**	0.42	0.05	0.29***

Adjusted *R*^2^ = 0.21; * *p* < 0.05; ** *p* < 0.01; *** *p* < 0.001

*ART* adherence to antiretroviral therapy; *CD4* cluster difference 4; *HIV* human immunodeficiency virus

## Discussion

Consistent with some previous research, older PLHIV had a higher QoL score. Some previous research speculated that older PLHIV had a higher level of acceptance of having been diagnosed with HIV [42, 43], were more likely turn to their religious faith, and were less likely to complain about their limitations [17]. On the other hand, other research indicated that younger PLHIV had a better QoL because of their better physical health [30]. In this research, after controlling for physical health, older PLHIV appeared to have a better QoL. Hence, it is likely that with improvements in care and therapy, older PLHIV would show improvements in QoL as well. In this research, employment had a positive influence on QoL. Such a relationship between employment and QoL was also identified in previous studies [15, 16]. Employment could provide additional financial resources and also improve social networking and social support [16]. All these factors could contribute to a higher QoL. However, to be employed, a person probably has to be healthier, and hence, already with a higher QoL. Rueda et.al [23] stated that the employment status had a stronger relationship with physical health than mental health. Future studies could use a longitudinal study design to assess the causal relationship between employment and QoL.

In this study, after controlling for other factors, personalized stigma, disclosure stigma, and self-image stigma were significantly associated with a lower QoL. Stigmatization and discrimination frequently prevent PLHIV from acquiring needed services and support. Misunderstanding of HIV/AIDS has probably led to stigmas and public fear. A recent study indicated that as high as 55% of the general public have a poor understanding of HIV/AIDS and tend to associate HIV/AIDS with female sex workers, men who have sex with men, and intravenous drug users, all of whom are subject to intense social stigmatization in Indonesia [44]. Such stigmatizing attitudes were also generally present among healthcare providers and varied by religious preferences [7]. Indonesia, the most populous Muslim-majority country, where cultural identity and self-awareness are based on religious faith [45], faces conflicting values regarding HIV/AIDS care and prevention. It is the government’s responsibility to provide needed care to PLHIV, educate the general public about scientific facts concerning HIV, and advocate protective behaviors, such as the use of condoms. Yet, efforts of public campaigns or health education within schools are frequently met with objections and resistance from conservative groups. Determining how to provide needed care, promote HIV prevention, and appease religious groups continues to be a challenge for the Health Ministry in Indonesia. Despite several public campaigns aimed at reducing the stigma associated with HIV/AIDS, their effectiveness has not been evaluated.

Access to healthcare services and medication adherence significantly contribute to the QoL among PLHIV, as indicated in this research and in other previous research [46, 47]. Similarly, those who had remained in an ART program for more than 1 year and those who had not missed their medications also tended to have a better QoL. In Indonesia, voluntary counseling and testing (VCT) and ART services can be obtained at primary health centers. VCT and ART service programs were first provided in 2005 and 2003, respectively. Both services have been provided free of charge since their initiation. In 2015, there were 2221 primary health centers that provided VCT and ART services. About 48% of primary care centers that provide VCT and ART services are located in Jakarta, East Java, and Papua Provinces where 45% of PLHIV are located.

Despite widespread access, the Ministry of Health reported only 41% of people with advanced HIV infection had received ART as of August 2018 [48]. Determining how to increase utilization rates of ART services among PLHIV has become a challenge for health officials. It was speculated that possible reasons for not receiving ART services include side effects, religious beliefs, stigmatization, difficulties incorporating medication moments in daily activities, a high pill burden, and difficulties accessing services [7, 8]. Since most ART service centers are located in urban areas, the additional costs of transportation and traveling times may deter some PLHIV living in rural areas from using those services. Improving access to services in rural areas would likely improve utilization rates. Since traditional values still discriminate against PLHIV, it is essential that their personal identity and privacy be protected throughout the process of receiving ART services. A willingness to use HIV-related services will hinge upon how well personal information is protected and how much PLHIV trust public systems to protect their identities. Thus far, no research has evaluated the level of trust among PLHIV regarding privacy protection. Moreover, evaluations regarding the protocols of service delivery, especially with regard to identity protection, should be conducted to increase the trust of PLHIV in public systems.

Although this study has contributed to understanding factors associated with the QoL among PLHIV in Indonesia, there are several limitations. First, only a limited number of variables were covered in this study, and other important factors related to QoL, particularly risky behaviors, the viral load, and the stage of infection, were not examined. Second, the cross-sectional nature of the analysis limited us from drawing causal inferences between explanatory factors and the QoL. And third, QoL might have been over-estimated, since participants were recruited only from ART clinics, and data were collected by clinic staffs. PLHIV receiving ART services were likely to have a higher level of QoL than those not receiving ART services. Although the use of clinic staffs to collect data may increase the willingness of PLHIV to participate in this study, it may also introduce possible bias in combination with the convenience sampling approach. Thus, our findings may have overestimated the level of wellbeing among PLHIV. Generalizing results to other areas in Indonesia should done with careful consideration of these issues. Despite these limitations, the study provides evidence of the importance of considering stigma subscales, social support, sociodemographic characteristics, and medication variables, in both research and practice, to promote the QoL among HIV patients in Indonesia.

## Conclusions

This study found that PLHIV of an older age, with a job, who live in an urban area, with better access to healthcare services, who are adherent to their medication, who are receiving ART treatment for > 1 year, and with better social support were positively associated with a better QoL. However, personalized stigma, disclosure stigma, and self-image stigma were associated with a lower QoL. Thus, promoting medication adherence for PLHIV and enhancing public awareness about HIV/AIDS in order to reduce stigmas and show support might positively impart a better QoL among PLHIV in Indonesia.

## Data Availability

Data from this study are available from the corresponding author upon reasonable request.

## Abbreviations

ACTG: AIDS clinical trial group
AIDS: Acquired immunodeficiency syndrome
ANOVA: Analysis of variance
ART: Antiretroviral therapy
CD4: Cluster of differentiation 4
HIV: Human immunodeficiency virus
MSPS: Multidimensional scale of perceived support
PLHIV: People living with HIV
QoL: Quality of life
SD: Standard deviation
VCT: Voluntary counseling and testing
WHOQOL-HIV BREF: World health organization quality of life in the HIV-infected person
